# Современные стратегии лечения ожирения у детей

**DOI:** 10.14341/probl13208

**Published:** 2022-12-20

**Authors:** О. В. Васюкова, П. Л. Окороков, О. Б. Безлепкина

**Affiliations:** Национальный медицинский центр эндокринологии; Национальный медицинский центр эндокринологии; Национальный медицинский центр эндокринологии

**Keywords:** диетотерапия, физическая активность, лираглутид, детское ожирение

## Abstract

Распространенность ожирения и связанных с ним метаболических нарушений у детей и подростков в Российской Федерации неуклонно растет, что требует от специалистов здравоохранения поиска новых методов лечения и профилактики. Лечение детского ожирения должно базироваться на комплексном подходе, включающем диетотерапию, расширение физической активности, поведенческую терапию и психологическое сопровождение. Для повышения эффективности формирования новых пищевых привычек и правильного пищевого поведения, а также с целью повышения приверженности детей и подростков к лечению используется медикаментозная терапия ожирения, направленная в первую очередь на снижение аппетита. Учитывая эффективность и безопасность аналогов глюкагоноподобного пептида 1 (лираглутида) у подростков, а также небольшое количество побочных эффектов со стороны желудочно-кишечного тракта, данный препарат является перспективным в комплексном лечении детского ожирения. В данном обзоре представлен анализ научной литературы по немедикаментозным и медикаментозным методам терапии ожирения у детей.

## Abstract (en)

The prevalence of obesity and related metabolic disorders in children and adolescents in the Russian Federation is steadily increasing, which requires healthcare professionals to search for new methods of treatment and prevention. The treatment of childhood obesity should be based on a comprehensive approach, including diet therapy, increased physical activity, behavioral therapy and psychological support. To increase the effectiveness of the formation of new eating habits and proper eating behavior, as well as to increase the adherence of children and adolescents to treatment, drug therapy of obesity is used, aimed primarily at reducing appetite. Considering the efficacy and safety of glucagon-like peptide 1 analog (Liraglutide) in adolescents, as well as a small number of gastrointestinal side effects, this drug is promising in the complex treatment of childhood obesity. This review presents an analysis of the literature on non-medicated and drug-based methods of treatment of childhood obesity.

## ПРОБЛЕМА ДЕТСКОГО ОЖИРЕНИЯ

Ожирение у детей и подростков в настоящее время является самым частым эндокринным заболеванием в России и мире. Несмотря на различные стратегии и предпринимаемые усилия, распространенность ожирения неуклонно растет [1]. Один из самых печальных трендов — увеличение ожирения среди детей младшего возраста (до 5 лет), а также рост числа морбидных форм в педиатрической популяции [2]. Опасность детского ожирения заключается в его недооценке как родителями и обществом в целом, так и врачами разных специальностей. Из века в век, на фоне войн, инфекционных эпидемий и голода пухлый малыш символизировал здоровье и благополучие. В ХХ в., в условиях избытка высококалорийной пищи и снижения повседневной активности, генетические основы «бережливого фенотипа» привели к быстрому распространению неинфекционной эпидемии — ожирения. Однако сознание общества, культовые традиции семьи и отдельных народов до настоящего времени ошибочно не дают усомниться в большей «успешности» для жизни пухлого ребенка. Как результат — позднее первичное обращение к детскому эндокринологу с уже выраженными, морбидными, метаболически осложненными формами заболевания. Но вместе с тем и среди врачебного сообщества до настоящего времени отсутствует единый подход к ребенку с избытком массы тела, и в первую очередь это «гиподиагностика» ожирения, о чем свидетельствуют низкие показатели выявляемости заболевания в нашей стране, по данным официальной статистики, по сравнению с результатами активных эпидемиологических исследований в отдельных регионах.

Другими словами, при отсутствии жалоб на избыточную массу тела данный диагноз зачастую не выставляется при обращении ребенка по поводу ОРЗ, ЛОР-патологии, нарушений менструального цикла, гастрита и др. Длительные наблюдения показали, что раннее начало ожирения связано с развитием сердечно-сосудистых и метаболических заболеваний, причем доклинические проявления возникают в подростковом возрасте и в конечном итоге приводят к увеличению смертности во взрослом возрасте [3][4]. Этому способствует и тот факт, что при отсутствии своевременного вмешательства детское ожирение «взрослеет» вместе с ребенком, приводя к развитию морбидных форм во взрослом состоянии [5]. Кроме того, ожирение зачастую сопровождается социальной стигматизацией и психологическими расстройствами, что, безусловно, ухудшает качество жизни детей, вплоть до полного отчуждения и самоизоляции. Поэтому ожирение у детей и подростков является серьезным, сложным и хроническим заболеванием, признанным таковым ведущими мировыми профессиональными сообществами [6][7]. И если мы признаем ожирение хроническим заболеванием, то каждый ребенок с ним должен быть диагностирован, и в каждом случае необходимо определить программу индивидуальной терапии. Оправданность такого вмешательства доказана в длительных наблюдательных исследованиях: уменьшение массы тела у детей с ожирением снижает риск развития сахарного диабета 2 типа, артериальной гипертензии, дислипидемии во взрослом состоянии [8][9]. Поэтому профилактика и лечение ожирения как можно раньше имеют первостепенное клиническое значение и социальную значимость.

В обзоре рассмотрены современные подходы к лечению детского ожирения. Проведен анализ отечественных и зарубежных публикаций в базе данных биомедицинских публикаций PubMed, Medline, eLibrary, EMBASE, Google Scholar и Cochrane library.

## МОДИФИКАЦИЯ ОБРАЗА ЖИЗНИ

Эффективное лечение детского ожирения возможно только при комплексном и длительном воздействии, направленном на модификацию образа жизни как самого ребенка, так и членов его семьи. Основу терапии ожирения составляют мероприятия по изменению рациона питания, расширению двигательной активности, а также психологическая коррекция, направленная на профилактику или лечение нарушений пищевого поведения.

Эффективность различных стратегий по модификации образа жизни у детей с ожирением оценена в многочисленных исследованиях и метаанализах [10–13]. Модификация образа жизни, основанная на школьных образовательных программах, в течение 6–72 мес приводит к снижению индекса массы тела (ИМТ) у детей с ожирением в среднем на 0,35 кг/м2 [10]. Анализ клинических данных более 23 тысяч детей в возрасте от 6 до 12 лет с ожирением, которым в течение 6 мес проводились обучающие мероприятия по формированию здорового образа жизни в образовательных учреждениях, продемонстрировал снижение ИМТ на 0,86 кг/м2 (95% доверительный интервал (ДИ) -1,59–-0,14) и уменьшение окружности талии на 3,2 см (95% ДИ -6,34–-0,07) [11]. В метаанализе H. Mandy и соавт., включившем 12 исследований, продемонстрировано, что снижение ИМТ у детей с ожирением на фоне модификации образа жизни в течение 6 мес составило 0,24 кг/м2 (95% ДИ -0,62–-0,14). Повышение уровня физической активности несет дополнительные преимущества в виде улучшения показателей метаболического профиля: повышения уровня холестерина липопротеинов высокой плотности, снижения уровня гликемии и инсулина натощак [12]. У детей с ожирением до 6-летнего возраста модификация образа жизни в течение 6–12 мес позволяет добиться снижения Z-критерия ИМТ на 0,3 (95% ДИ -0,4–-0,2) в течение первого года; на 0,4 (95% ДИ -0,6–-0,2) — в течение 12–18 мес; на 0,3 (95% ДИ -0,4–-0,1) в течение 2 лет [13].

## ПСИХОЛОГИЧЕСКОЕ СОПРОВОЖДЕНИЕ

Большинство детей и подростков с ожирением и избыточной массой тела не готовы инициировать изменения в образе жизни и пищевом поведении в связи с наличием нарушений в эмоционально-волевой сфере, повышенным уровнем тревожности, раздражительностью и лабильностью настроения. Стимулом к приему пищи зачастую являются не голод, а тревога, плохое настроение, разочарование, обида, одиночество или скука. Важную роль в развитии ожирения играют негативные эмоции, возникающие у детей в результате межличностных отношений в школе, семье, среди друзей: чувство вины, раздражения, злости, беспомощности, обескураженности, разочарования, ревности. При анализе эмоционального статуса пациентов с ожирением отмечено, что они испытывают большие трудности с выражением эмоций (алекситимия) [14]. Также обращают на себя внимание такие психологические особенности, как импульсивность, склонность к категоричному мышлению и неудовлетворенность образом тела [15].

Таким образом, медико-психологическое консультирование и сопровождение являются важнейшими факторами, обеспечивающими эффективность любых стратегий по модификации образа жизни и снижению веса. Психологическая диагностика и сопровождение детей с ожирением помогают своевременно выявлять и корректировать отклонения в эмоциональной сфере, формировать и поддерживать мотивацию. Одной из основных проблем в лечении ожирения является неспособность пациентов удерживать достигнутые результаты в уменьшении веса, срывы и постепенный обратный набор веса. Наиболее частыми психологическими причинами этого являются нарушение способности к длительным систематическим усилиям и недостаточная приверженность к новому образу жизни.

К наиболее распространенным методам психологической коррекции относятся когнитивно-поведенческая терапия, коррекция психоэмоционального дисбаланса, коррекция пищевого поведения (коррекция привычки), обучение стратегиям, направленным на удержание результата, гештальт-терапия. Учитывая важнейшую роль семьи в формировании здорового образа жизни, современные стратегии лечения детского ожирения включают семейные поведенческие программы [16][17]. Так, вработе D.E. Wilfley и соавт. активная психологическая и социальная поддержка семьи способствовала достижению клинически значимого изменения массы тела у детей по сравнению с контрольной группой [17].

## ДИЕТОТЕРАПИЯ

Современное направление диетотерапии при ожирении включает нормокалорийное питание, сбалансированное по всем нутриентам [18]. Диета не должна нарушать физическое и психическое развитие ребенка, препятствовать выполнению физических нагрузок, а рацион должен обеспечивать достаточное насыщение и при этом быть вкусным и разнообразным. Оптимальный для детей и подростков суточный рацион должен состоять из 3 основных приемов пищи с горячим блюдом и 1–2 дополнительных (второй завтрак, полдник). Распределять приемы пищи необходимо с учетом особенностей режима дня (занятия в школе, спортивные секции, кружки и т.д.). Подходы должны быть индивидуальными и гибкими. По энергетической ценности завтрак должен составлять 20–25% суточной энергетической потребности, обед — 30–35%, ужин — 15–20%. На дополнительные приемы пищи выделяется: на второй завтрак — 5–10% суточного количества калорий, на полдник — 10–15%. Основные приемы пищи должны обеспечить достаточное поступление макронутриентов: белков, жиров и углеводов. На завтрак следует готовить кашу, блюда из яиц, творог, горячий напиток. Обед должен включать 3–4 блюда: салат, суп, второе (с обязательным включением мяса или рыбы), напиток. Полдник обычно состоит из молочного продукта (творожное блюдо, йогурт, кефир и др.), хлебобулочного изделия или выпечки, фруктов или ягод. Ужин обязательно должен включать горячее блюдо (овощное, крупяное, творожное и т.п.) и горячий напиток. В качестве перекуса детям можно рекомендовать фрукты, сухофрукты, орехи (в небольшом количестве), кисломолочные напитки (йогурт питьевой, кефир, ряженку и др.), зерновые батончики, смузи, хлебцы. Наиболее подходящими способами приготовления пищи являются отваривание, тушение, запекание, приготовление на пару [19].

Строгие диетические ограничения могут приводить к формированию нарушений пищевого поведения по ограничительному и экстернальному типам, поэтому изменения в питании детей следует вводить постепенно с активным использованием психологической поддержки при необходимости.

## ФИЗИЧЕСКАЯ АКТИВНОСТЬ

Регулярная физическая активность необходима для эффективного контроля массы тела и профилактики избыточной массы тела и ожирения в детском возрасте. Поддержание достаточной двигательной активности ассоциировано с множественными благоприятными эффектами (улучшение композиционного состава тела за счет увеличения количества тощей массы, снижение артериального давления, снижение метаболических рисков, повышение мышечной силы и увеличение массы костной ткани). Согласно данным метаанализа 22 исследований, при повышении двигательной активности у взрослых значительно снижается риск общей смертности. Так 2,5 ч умеренной физической активности в неделю снижают риск общей смертности на 19%, 7,5 ч в неделю — на 24% [20].

Детям от 6 до 17 лет необходимы ежедневные физические нагрузки умеренной и высокой интенсивности в общей сложности не менее 60 мин. Физические нагрузки высокой интенсивности рекомендовано включать в обязательный час ежедневной физической активности и выполнять не менее 3 раз в неделю [18][21]. Физическая активность свыше 60 мин в день дает дополнительные преимущества для здоровья. Рекомендованная ежедневная продолжительность физических нагрузок (60 мин и более) может складываться в течение дня из более коротких нагрузок (например, 2 раза в день по 30 мин).

К сожалению, лишь четверть подростков и треть детей дошкольного и младшего школьного возраста соблюдает данные рекомендации. Результаты исследования HBSC (Health Behavior in School-age Children) демонстрируют, что рекомендованных 60 мин ежедневной двигательной активности достигают лишь 23,1% мальчиков и 14% девочек в возрасте 13–15 лет [22].

В исследовании IDEFICS, включившем детей в возрасте до 2 до 10 лет из 9 европейских стран, показано, что рекомендованный ВОЗ уровень двигательной активности достигается у 2,0–14,7% девочек и 9,5–34,1% мальчиков [23].

Для детей до 5-летнего возраста разработаны отдельные дефиниции по характеру и продолжительности физической активности, направленные на профилактику гиподинамии в раннем детском возрасте. Под физической активностью для детей младшего возраста подразумеваются различные игры: например, лежа на полу, с игрушками, ползание, гимнастика для малышей и т.д. Если ребенок еще не может ползать, рекомендуется проводить не меньше 30 мин в день лежа на животе. Для детей в возрасте 1–4 лет рекомендуются различные виды двигательной активности любой интенсивности в общей сложности не менее 180 мин в день для профилактики избыточной массы тела и ожирения [24].

## МЕДИКАМЕНТОЗНАЯ ТЕРАПИЯ

К сожалению, подавляющее большинство детей с ожирением имеют низкую мотивацию к снижению веса и поддержку со стороны членов семьи, что клинически проявляется краткосрочным снижением массы тела с последующим «рикошетным» набором веса. В таких случаях для повышения эффективности используется фармакотерапия, назначаемая в дополнение к модификации образа жизни и обеспечивающая снижение массы тела, а также улучшающая прогноз в отношении развития осложнений ожирения и ассоциированных метаболических нарушений. Критериями инициации медикаментозной терапии ожирения у детей являются: возраст не менее 12 лет, неэффективность мероприятий, направленных на формирование здорового образа жизни, продолжительностью не менее 1 года [18]. К препаратам, зарегистрированным и разрешенным к применению в РФ при детском ожирении, в настоящее время относятся орлистат и лираглутид [18].

Орлистат является ингибитором желудочной и панкреатической липаз, которые участвуют в гидролизе триглицеридов и необходимы для абсорбции жиров в тонком кишечнике. В результате действия препарата нарушается расщепление пищевых жиров и уменьшается их всасывание, что создает дефицит энергии и приводит к снижению массы тела. Препарат способствует уменьшению гиперхолестеринемии путем снижения количества свободных жирных кислот и моноглицеридов в просвете кишечника, что уменьшает растворимость и последующее всасывание холестерина вне зависимости от степени снижения массы тела. Наиболее частыми побочными эффектами терапии являются боль или спазмы в животе, газообразование, стеаторея, возникающие в результате прямого действия препарата. Выраженность побочных эффектов связана с приверженностью пациента к диетотерапии, направленной на ограничение употребления пищи, богатой жирами. Средняя терапевтическая доза препарата составляет 120 мг 3 раза в сутки во время или после еды. В метаанализе, включившем 779 подростков из трех исследований в возрасте 12–18 лет со средним исходным ИМТ 37,4 кг/м2, выявлено лишь незначительное снижение ИМТ у пациентов групп орлистата (от -0,94 до -0,50 кг/м2) [25]. В результате длительного лечения орлистатом возможно развитие дефицита жирорастворимых витаминов и минералов, что может потребовать дополнительной саплементации дефицитных микронутриентов. Из-за высокой частоты развития гастроинтестинальных побочных эффектов у подростков, посещающих школу, где может быть ограничено пользование туалетом, орлистат не считается препаратом 1-й линии для лечения ожирения у подростков. В настоящее время не представлены долгосрочные клинические исследования изменения метаболических рисков на фоне приема орлистата.

Лираглутид является аналогом глюкагоноподобного пептида 1 (ГПП-1). ГПП-1 секретируется L-клетками подвздошной кишки и толстого кишечника в ответ на стимуляцию перевариваемой пищей и усиливает секрецию инсулина по глюкозозависимому механизму, т.е. действует как инкретин. Рецепторы ГПП-1 (ГПП-1Р) представлены в клетках поджелудочной железы, легких, сердце, желудочно-кишечном тракте, жировой ткани, а также в центральной нервной системе. Основными регионами, экспрессирующими ГПП-1Р в головном мозге, являются кора, гипоталамус (дугообразные, паравентрикулярные ядра, вентромедиальная зона), таламус, миндалина, бледный шар, отвечающие в том числе за регуляцию энергетического баланса и пищевое поведение [26]. Введение ГПП-1 снижает аппетит и приводит к уменьшению потребления пищи. ГПП-1 также замедляет скорость опорожнения желудка и подавляет секрецию соляной кислоты благодаря паракринной активации ГПП-1Р, локализованных на афферентных волокнах блуждающего нерва. Периферические ветви афферентных волокон n. vagus, иннервирующие желудок и кишечник, посредством механо- и хеморецепторов обеспечивают мониторинг объема и характера нутриентов. Центральные терминали доставляют эту информацию в ствол головного мозга и далее — нейронами ядер одиночного тракта к гипоталамусу, который генерирует нисходящие импульсы, стимулирующие или ингибирующие моторные волокна блуждающего нерва, достигающие желудка и кишечника [27].

Недавние исследования продемонстрировали, что ГПП-1 вырабатывается в небольших количествах в нейронах луковицы обонятельного нерва и вкусовых клетках полости рта, что может модулировать обонятельное и вкусовое восприятие пищи ивносить дополнительный вклад в снижение аппетита [28][29]. Механизм действия нативного ГПП-1 и лираглутида на массу тела имеет некоторые отличия. Прежде всего препарат обладает преимущественно центральным действием. Лираглутид снижает активность кортиколимбических структур (париетальной коры, инсулы и подушки), обеспечивающих гедонистический контроль пищевого поведения. С учетом функций данных отделов препарат, соответственно, подавляет внимание к особо вкусной пище, восприятие ее привлекательности (гедонистической ценности) и мотивацию к ее приему [30]. Лираглутид оказывает минимальное влияние на моторику желудка. В исследовании J. van Can и соавт. у лиц с ожирением замедление опорожнения желудка наблюдалось лишь в течение 1-го часа после введения препарата, уже через 5 ч разница по сравнению с контрольной группой, получавшей плацебо, не прослеживалась [31]. Таким образом, влияние лираглутида наэнергетический баланс связано преимущественно со снижением потребления энергии. Лираглутид повышает чувство насыщения и сытости после еды и уменьшает выраженность голода, что в итоге и приводит к уменьшению потребления пищи и формированию «отрицательного энергетического баланса».

Эффективность и безопасность лираглутида в дозе 3 мг в сутки у подростков 12–18 лет изучены в международном рандомизированном контролируемом двойном слепом исследовании SCALE TEENS [32]. Исходно все подростки (125 человек в группе лираглутида и 126 в группе плацебо) были сопоставимы по возрасту (средний 14,5 года), полу, стадии полового развития, имели выраженное ожирение (SDS ИМТ 3,14–3,2) и низкий ответ на предшествующую модификацию образа жизни. Через год терапии (лираглутид 3,0 мг или плацебо подкожно 1 раз в день) в дополнение к изменению образа жизни лираглутид снижал SDS ИМТ больше (на 0,25), чем плацебо (0,02), что соответствовало изменению ИМТ на -0,22 (95% ДИ -0,37–-0,08; P=0,002), или -4,5 кг в абсолютных значениях [32]. На фоне отмены препарата на протяжении 26 нед наблюдения отмечалось обратное увеличение массы тела. Итоговое изменение SDS ИМТ составило -0,06 в группе лираглутида и +0,07 в группе плацебо. Большее число пациентов на фоне лираглутида имели гастроинтестинальные побочные эффекты в виде тошноты и рвоты в сравнении с плацебо (64,8% vs. 36,5%). Однако большинство побочных эффектов носило нетяжелый и преходящий характер и было связано с титрацией дозы в начале терапии: доза увеличивалась еженедельно с 0,6 мг до 3,0 мг или максимально переносимой дозы. В случае непереносимости доза могла быть снижена до предыдущего уровня. В результате 82% подростков достигли целевой дозы лираглутида 3 мг и удерживали ее в течение всего периода лечения. Отказ от терапии в связи с развитием побочных эффектов зарегистрирован у 4% пациентов группы лираглутида и у 2,4% в группе плацебо.

В случае развития тошноты и/или рвоты у пациента на фоне терапии лираглутидом необходимо проанализировать фактическое питание: зачастую пациенты, несмотря на обучение по модификации образа жизни, нарушают данные рекомендации, что может провоцировать развитие побочных эффектов терапии [33–35]. Лираглутид способствует более быстрому насыщению. Поэтому пациента/семью следует предупредить о необходимости пропускать прием пищи при отсутствии голода; но при этом стараться всегда завтракать; уменьшать ранее привычную порцию вдвое — съесть вторую половину через 10–20 минут после первой только в том случае, если сохраняется чувство голода; избегать пищи с высоким содержанием жиров и«привычно» больших объемов, избегать нахождения в условиях приготовления пищи, если запахи ухудшают самочувствие. Кроме этого, необходимо напоминать о достаточном потреблении питьевой воды и приоритетном выборе свежеприготовленной пищи. В некоторых случаях могут потребоваться снижение дозы лираглутида до предыдущей и/или симптоматическое назначение лекарственных средств. При развитии диареи рекомендуется уменьшить потребление молока и молочных продуктов, в случае запора — увеличить потребление клетчатки. Вопрос о назначении симптоматической терапии (энтеросорбенты, противорвотные, противодиарейные препараты) решается индивидуально. Если после 12 нед применения препарата в дозе 3 мг в сутки потеря в массе тела составляет менее 4% ИМТ или z-показателя ИМТ, терапию следует прекратить или пересмотреть. Учитывая наличие центрального механизма действия лираглутида, необходим контроль психологического состояния подростка. Согласно данным исследования SCALE TEENS, 10,4% подростков группы лираглутида и 14,3% группы плацебо характеризовались наличием нарушений в виде снижения настроения, депрессии, нарушения сна. Вместе с тем 1 пациент группы лираглутида, ранее наблюдавшийся по поводу синдрома дефицита внимания с гиперактивностью, совершил суицид, признанный исследователями как не связанный с терапией, и 2 пациента в группе плацебо сообщили о попытках суицида. Это лишний раз подчеркивает уязвимость подростков и необходимость мультидисциплинарного подхода с привлечением психолога, психотерапевта в терапии ожирения. В рутинной практике следует уделять внимание психологическому состоянию ребенка путем уточнения жалоб или применения опросников, в случае выявления признаков депрессивного расстройства своевременно привлекать специалиста. Суммарный эффект терапии лираглутидом 3 мг в виде снижения массы тела, стагнации SDS ИМТ, уменьшения полифагии и доказанной безопасности оправдывает назначение препарата даже при небольшой степени ожирения с целью профилактики прогрессирования заболевания при условии комплаентности подростка и семьи, а также приверженности мерам по модификации образа жизни.

## ЗАКЛЮЧЕНИЕ

Современные стратегии лечения детского ожирения должны быть долгосрочными и базироваться на комплексном подходе, направленном на изменение рациона питания и пищевых привычек, расширение двигательной активности как самого ребенка, так и других членов семьи. Психологическое сопровождение необходимо для профилактики нарушений пищевого поведения или коррекции нарушений эмоционально-волевой сферы. Медикаментозная терапия ожирения, направленная на снижение аппетита, в сочетании с модификацией образа жизни может значительно повысить эффективность программ по снижению и контролю веса у детей и подростков с ожирением.

## ДОПОЛНИТЕЛЬНАЯ ИНФОРМАЦИЯ

Источник финансирования. Работа выполнена по инициативе авторов без привлечения финансирования.

Конфликт интересов. Авторы декларируют отсутствие явных и потенциальных конфликтов интересов, связанных с публикацией настоящей статьи.

Участие авторов. Васюкова О.В. — написание и редактирование статьи; Окороков П.Л. — написание статьи; Безлепкина О.Б. — редактирование и утверждение финальной версии статьи. Все авторы одобрили финальную версию статьи перед публикацией, выразили согласие нести ответственность за все аспекты работы, подразумевающую надлежащее изучение и решение вопросов, связанных с точностью или добросовестностью любой части работы.
